# Effectiveness of Different Pain Control Methods in Patients with Knee Osteoarthritis after Knee Arthroplasty

**DOI:** 10.1155/2017/8738924

**Published:** 2017-06-19

**Authors:** Jin-Lain Ming, Shiao-Pei Hung, Hui-Mei Huang, Li-Hua Tseng, Chiung-Hui Huang, I-Chun Chen, Yu-Ping Chen, Chien-Kun Ting, Chen-Yin Tung

**Affiliations:** ^1^Department of Nursing, Taipei Veterans General Hospital, No. 201, Section 2, Shipai Road, Taipei 112, Taiwan; ^2^Department of Health Promotion and Health Education, National Taiwan Normal University, No. 162, Section 1, Heping E. Road, Taipei 106, Taiwan; ^3^Department of Anesthesiology, Taipei Veterans General Hospital and National Yang-Ming University, No. 201, Section 2, Shipai Road, Taipei 112, Taiwan

## Abstract

The number of patients with knee osteoarthritis has increased in tandem with population aging. Consequently, the number of knee arthroplasties has also risen. The postoperative pain is the biggest challenge faced by patients soon after knee arthroplasty; therefore, this study is among different methods for post-knee arthroplasty pain control. A prospective longitudinal research design was employed; 177 adult patients who proposed for primary knee arthroplasty were enrolled and recruited. The patients were divided into conventional Group 1 (*n* = 120) and patient-controlled analgesia (PCA) Group 2 (*n* = 57) according to the treatment methods they received. All patients experience the highest pain level on the day of their surgery; women complained of higher pain levels than men did, while the PCA group had lower postoperative pain. Meanwhile, patients with general anesthesia experienced more pain than those with spinal anesthesia in postoperative period. Patients with a higher postoperative pain index have a smaller optimal knee flexion angle. The PCA group had lower postoperative pain; all patients experienced the highest pain level on the day of their surgery. The results of this study could serve as a reference for nurses where PCA ensures a better postoperative pain control and therefore facilitates recovery and improves the quality of nursing.

## 1. Introduction

Knee osteoarthritis which is common has a great impact of the life quality because of the joint pain and the consequently physical dysfunction [1]. With the global population aging, the number of patients with knee osteoarthritis has increased as a consequence [2]. Knee arthroplasty is a common treatment for knee osteoarthritis [3], with over 30,000 people who underwent knee arthroplasty each year according to statistics compiled by the Taiwan Ministry of Health and Welfare [4]. Therefore, postoperative knee arthroplasty care has become a noteworthy heath concern among contemporary aging societies.

Patients require analgesics soon after undergoing knee arthroplasty. Opioid analgesics are the first choices for postoperative pain relief and are conventionally administered through intravenous or intramuscular injection [5]. With the advance in medical technologies, patient-controlled analgesia (PCA) has become an alternative pain control method.

The aim of this study is to compare the effectiveness of PCA with conventional nurse-based intravenous or intramuscular opioid pain control. The postoperative knee functions referred to as the knee flexion angle will also be evaluated.

## 2. Material and Methods

This prospective study used a longitudinal research design and the participants were divided into two groups according to their pain control methods. Group 1 consisted of post-knee arthroplasty patients who were given analgesics through intravenous injection by nurses following doctors' order when patients experienced wound pain; Group 2 consisted of patients on whom PCA was employed for administering analgesics through intravenous or epidural injection. PCA regimens were prepared according the expense of IV or Epi PCA of the individual. The IVPCA formulation contained Morphine 100 mg while Epi PCA formulation contained 0.1% Marcaine + Fentanyl 500 *μ*g. The infusion dose was determined by the anesthesiologist according to the patient's age and body weight. No additional rescue dosage analgesics were given by nurses. Inclusion criteria of this study include patients aged 20 years and above who underwent unilateral knee arthroplasty for the first time, being able to communicate through speech or text, and willingness to participate in this study. The patient should be excluded from this study if they are unconscious or unable to communicate through any linguistic means or they underwent knee arthroplasty because of trauma or receiving bilateral knee arthroplasty.

This study was approved by the institutional review board of Taipei Veterans General Hospital (2015-02-002CC). Participants were recruited from April to November 2015. The researchers visited patients 1 day before surgery, explained the research purpose and methods and subsequent research procedures, and acquired their written informed consent and conducted data collection. From all patients the following data were collected: demographics, surgical pain cognition and attitude, and preoperative pain level, and knee function data was collected on the day before surgery, during time spent in the recovery room, and after patients returned to their wards; postoperative pain level data was collected from the first day until the third day after surgery with knee function data collected simultaneously.

The Pain and Opioids Analgesics Beliefs Scale–Surgical Pain Version (POABS–SP) developed by Lee et al. [6] was used to assess individuals' postoperative pain and their 16 beliefs on the use of narcotic analgesics. The scale consisted of five items on pain tolerance, six items on the negative effects of analgesics, and five items on the nature of pain and analgesics. A five-point Likert scale was employed (0 =* strongly disagree*; 4 =* strongly agree*). A higher score meant a higher negative belief regarding postoperative pain and the application of narcotic analgesics. The scale had high reliability and validity, displaying internal consistency reliability (Cronbach's *α* = 0.75).

Pain level assessment was done by a pain numerical rating scale (NRS) which is recommended by The* Clinical Practice Guideline: Nursing Care of Postoperative Pain* published by the Taiwan Nurses Association. Pain intensity is divided into 10 levels (0–10), whereby “0” represents painlessness and “10” represents the most severe pain imaginable. The ratings are assigned by patients according to their pain intensity. The scale evaluated the lowest degree of pain when the patient rested and the highest degree of pain when the patient was active [7].

Knee functions referring to the knee flexion angle were also evaluated for each patient. The measured variable was based on the flexion angle of patients' knees when they were conducting passive motion. The knee flexion angles were measured using a continuous passive motion.

### 2.1. Statistical Analysis

Data entry and analysis were conducted using the Chinese version of the SPSS 18.0 software package. The statistical methods employed included frequency distribution, percentage, univariate analysis, and a generalized estimating equation (GEE) model. Anesthesia method is selected by anesthesiologists, where the anesthesia may also be one of the factors that affect the postoperative pain score. By GEE analysis, we try to perform subgroup comparison between the two groups of different types of anesthesia with different time pain scores.

## 3. Results

**Figure 1:**
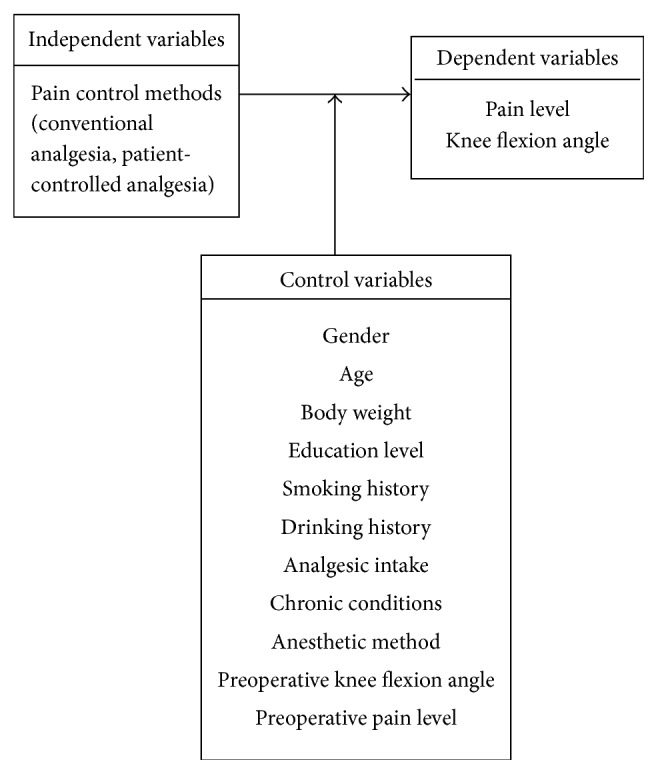
Research framework.

A total of 177 participants were recruited in this study, with 120 assigned to Group 1, which had a female majority (76 patients, 63.3%). Among members of Group 1, the average age was 71 ± 9 years, 75% had surgical experience, the preoperative average pain (NRS) was 2 ± 2, the preoperative highest pain level was 5 ± 3, and the preoperative knee flexion angle was 112° ± 18°. Group 2 had 57 participants, with a similar female majority of 46 (80.7%) participants and an average age of 73 ± 8 years (see Table 1 for a comparison of demographic data between the two groups). The POABS scores for the conventional and PCA groups were 41 ± 7 and 38 ± 8, respectively (Table 2). The samples in the two groups likely possessed distinct characteristics, such as gender, age, education level, smoking history, drinking history, and chronic diseases. These were viewed as control variables when the differences between the two groups were compared (Figure 1).

### 3.1. Pain Variation Analysis in relation to Different Pain Treatment Methods

*(1) Difference in Lowest Pain Ratings*. GEE was used to investigate the difference in the lowest pain rating, whereby 13 independent variables including age, gender, weight, surgical site, anesthetic method, surgical experience, analgesic intake, drinking habits, smoking history, chronic disease history, chronic disease history, surgical pain, and analgesic beliefs were subjected to univariate analysis. Gender, anesthetic method, surgical pain, and analgesic beliefs attained significance (all *p* < .05). Therefore, these four variables were controlled in the GEE model, which revealed that the lowest pain rating of women was significantly higher (0.304) than that of men. After controlling the moderating variables, results of the GEE model analysis showed no difference in the lowest pain rating between the conventional and PCA groups. However, the rating difference rose to 3.092 when participants returned to their wards from the recovery room; the rating difference between postoperative day 1 and that of the recovery room was 1.425, and the difference between postoperative day 2 and that of the recovery room was 0.542; the differences were all significant. The results again displayed a significant difference when the model was adjusted for the interaction between time and group, whereby the lowest pain rating of Group 2 was 0.846 lower than that of Group 1 when participants returned to their wards from the recovery room (Table 3).

**Figure 2:**
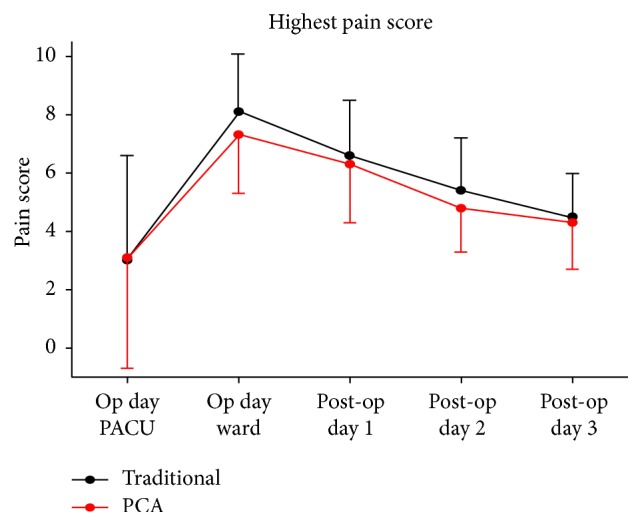
Distribution of the highest pain rating between the two groups.

*(2) Difference in Highest Pain Ratings*. The highest pain ratings of Group 1 were 3.0 (recovery room), 8.1 (ward), 6.6 (postoperative day 1), 5.4 (postoperative day 2), and 4.5 (postoperative day 3). The highest pain ratings of Group 2 were 3.1 (recovery room), 7.3 (ward), 6.3 (postoperative day 1), 4.8 (postoperative day 2), and 4.3 (postoperative day 3) (Table 4) (Figure 2). The pain ratings for both groups peaked when the patients returned to their wards on the day of surgery, followed by a daily decline in value. The pain rating of Group 1 was higher than that of Group 2 from the day of the surgery to postoperative day 3. GEE was used to investigate the variance of the highest NRS scores of the two groups at different times. All 13 aforementioned independent variables were first subjected to a univariate analysis, in which gender, anesthetic methods, surgical pain, and analgesic beliefs achieved significant differences (*p* < .05). Accordingly, they were controlled in the GEE model, which revealed a significant difference for gender and anesthetic methods; the highest pain rating of women was 0.444 higher than that of men, and it was 1.124 higher for participants who underwent general anesthesia than for those who underwent spinal anesthesia. After controlling for the moderator variables in the GEE model analysis, no difference was detected in the highest pain ratings of the two groups. However, the difference rose to 5.058 when the participants returned to their wards from the recovery room; the difference between postoperative day 1 and that of the recovery room was 3.558; the difference between postoperative day 2 and that of the recovery room was 2.35; and the difference between postoperative day 3 and that of the recovery room was 1.442; the differences were all significant. However, these results became nonsignificant when the model was adjusted for the interaction between time and group. In other words, the highest pain rating did not change according to the different analgesic methods applied and time (Table 5).

### 3.2. Knee Function Variation Analysis in relation to Different Pain Treatment Methods

This study investigated postoperative knee function variation by measuring participants' optimal knee flexion angle for postoperative days 1–3. The optimal knee flexion angles of Group 1 were as follows: 75° (postoperative day 1), 88° (postoperative day 2), and 95° (postoperative day 3). The optimal knee flexion angles of Group 2 were as follows: 68° (postoperative day 1), 80° (postoperative day 2), and 90° (postoperative day 3) (Table 6). The optimal knee flexion angles of both groups improved daily, with Group 1 showing a superior optimal flexion angle compared to the PCA group. Using GEE to investigate the difference of the optimal knee flexion angle on different times between the two groups, all aforementioned 13 independent variables were subjected to a univariate analysis. Gender, age, BMI, analgesia intake, preoperative optimal knee flexion angle, and the highest postoperative pain NRS demonstrated significant differences (all *p* < .05). Thus, they were controlled in the GEE model, which revealed that the highest postoperative pain rating still displayed a significant difference, whereby the optimal knee flexion angle decreased by 1.331° when the highest pain rating increased by 1. After controlling the moderating variables in the GEE model analysis, no significant difference in the optimal knee flexion angle was discovered between the two groups. However, postoperative day 2 showed an 11.6° increase compared to the previous day, and postoperative day 3 had an 18.37° increase compared to postoperative day 1. In other words, flexion angle of knee increased day by day (*p* < .001); no significant differences were noted between the two groups (*p* = .195). The postoperative pain score also affected knee flexion angle (*p* = .004) (Table 7).

### 3.3. Subgroup of Anesthetic Method in relation to Different Pain Treatment Methods

By clinical experience, type of anesthesia played an important role in the postoperative pain very much; further subgroup analyses were needed to see how and how much these factors influence. There are 111 (82.5%) who received SA and 9 who received GA (17.5%) in Group 1 while 49 (85.96%) and 8 (14.04%) received SA and GA in Group 2 consequently (Table 8). No significant difference was noted for different anesthesia type between the two groups (*p* = .168), while GEE statistical analysis was applied to test the pain scores of different anesthesia and different time. With the increasing of number of days after surgery, the lowest pain fraction will drop; there is no significant difference found in the third day after surgery and there is also no difference under the different anesthesia (Table 9). With the increasing of number of days after surgery, the highest pain scores will decline. In the day of surgery, pain score is 4.763 points higher than that in the recovery room. In the first day after surgery, it is 3.429 points higher and 2.141 and 1.339 higher for the second and third days after surgery. They were all significantly higher than that in the recovery room (*p* < .001). Patient receiving GA have a 1.053 higher pain score than those who received SA (*p* = .004) (Table 10).

The highest painful scores appeared on the day of surgery after the patients were sent back to ward from recovery room, and the degree of pain decreased with the increasing of number of days. Anesthesia methods affected the highest pain score. The anesthesia type also influenced the angle of flexion, the angle increasing day by days after operation (*p* < .001). It is not affected by the difference anesthesia types (*p* = .631) (Table 11).

## 4. Discussion

This study used a prospective longitudinal research design to investigate the effectiveness of different pain treatment methods (conventional analgesia and PCA) in patients who underwent knee arthroplasty. The results indicated that in terms of the lowest and highest pain scores the pain intensities of both groups were at their peaks when participants returned to their wards on the days of their surgeries, followed by a daily decline. Group 2 had a lower pain intensity compared to Group 1; women reported greater pain than men; patients who were more concerned about surgical pain and analgesia had a lower average pain score after surgery than did those who were less concerned; and overall pain level ranged from moderate to severe from the day of surgery to postoperative day 2. Knee bending angle increases after surgery; pain scores will lead to knee flexion angle.

Generally higher pain sensitivity among women, together with psychological factors such as higher levels of anxiety and depression, causes them to experience higher pain levels compared to men. A study by Pope et al. [8] revealed that women experience significantly higher pain levels within 24–48 hours after receiving knee arthroplasty. Akyol et al. [9] reached a similar conclusion. There are also studies showing that degree of cell activity is different between sexes; women tend to feel more pain than men [10]. Tverskoy et al. concluded that different types of anesthesia will affect the highest postoperative pain scores after inguinal herniorrhaphy; patients receiving spinal anesthesia have less pain score than those receiving general anesthesia [11]. It is similar to our results.

When lowest pain ratings were compared, the pain experienced by both groups in this study was at its highest level when the participants returned to their wards on the day of their surgeries, after which a gradual, daily decline in pain was observed. The pain level of Group 1 was higher than Group 2 surgical day. Choi et al. conducted a meta-analysis systemic review about effectiveness of epidural PCA; four randomized controlled trials were included [12]. Their results showed that the epidural PCA method significantly relieves early postoperative pain (4 to 6 hours after surgery) (95% CI: −1.30, −0.02). The surgery degree of pain was noted when patient was sent back to the ward from PCAU; this may be because no more anesthesia exists and thus highlights the use of PCA in this period. PCA's effect of pain relief is better than the traditional methods.

No difference in the highest pain ratings of the two groups was found with time on analgesic method. Both groups experienced severe pain: in the case of Group 1, when they returned to their wards on the days of their surgeries as well as on postoperative day one (NRS value = 7-8); in the case of the PCA group, when they returned to their wards on their days of surgeries (NRS value = 7). Our results are similar to that of the study by Akyol et al. [9]. Nurses may lose track of patients' pain patterns because of various interruptions that occur throughout their work, leading to inadequate assessment and treatment strategies [13]. Accordingly, this concern should be given more attention and handled appropriately.

Knee flexion function after surgery gradually increased; pain scores will lead to knee flexion angle. Research support provides better pain relief, leading to faster postoperative knee rehabilitation [14]. One possible factor was the intervention measures such as preoperative health education that were provided to the PCA group, leading to their superior performance in knee flexion angle and the functional assessment scale, thus displaying a significant difference [15]. First-line nursing personnel should be able to know pain problems quickly and give appropriate care. The primary aim of nursing staff [16] is to have a pain management protocol that provides patient-centered pain management with patient and his/her families [17]. A pain management protocol begins with a comprehensive pain assessment to understand the patient's experience and treatment goals, followed by the selection of suitable pain assessment instruments and provision of appropriate treatment. Follow-ups on pain assessment are conducted to monitor related side effects [18] and improve the quality of postoperative knee arthroplasty care. There are still several limitations in this study. First, this study was limited to patients with knee osteoarthritis who underwent knee arthroplasty at a medical center in Taipei. Therefore, the results may not be generalized to patients in other medical divisions or those who underwent different forms of arthroplasty. Second, because postoperative pain and functional recovery are influenced by numerous factors, the research framework investigated the effectiveness of post-knee arthroplasty pain control according to analgesic methods, basic attributes, and illness conditions. Other noteworthy variables such as psychological state and differences in support systems can be included in future research designs. Third, because research data collection was terminated after postoperative day 3, this study was unable to investigate the influence of long-term functional recovery after the surgery. However, the results can serve as a basis for the future development of systematic pain care intervention, providing references for clinical nurses and improving the quality of pain care, thus achieving the goal of pain-free hospitals. Fourth, although the questionnaire employed in this study had high reliability and validity, Group 2 had a lower-case than Group 1 did, and the monitoring of objective physiological indicators such as heart rate and blood pressure was not included in the assessment of the results. Future studies should use assessment tools that encompass both subjective and objective aspects to ensure more accurate measurements. The results of this study can be used as clinical guide for nursing staff to care for patients after knee surgery especially for those worried about postoperative pain and narcotic analgesic drugs. It can also be applied in patient education before surgery to reduce patient anxiety as well as providing several different analgesic options. It may thus improve the quality of surgery throughout the perioperative period care. Fourth, as for the research tools, in this study, the questionnaire survey, although it was with good reliability and validity, it was with the relative small sample size of self-control group. The results also do not include the parameters of the physiological indicators from monitors, such as heart rate and blood pressure; they could be better assessment tools for more accuracy and effectiveness in the future.

In conclusion, the results of this study showed pain scores reduced with the increasing of number of days after surgery in patients with arthroplasty. Knee flexion angle also increases with the increasing of number of days after surgery, while the pain scores were lower. The pain score in PCA group is lower than the traditional group, and different ways of anesthesia will also affect the degree of postoperative pain.

## Figures and Tables

**Table 1:** Demographic characteristics of participants (*N *= 177).

Variables	Group 1 (*n* = 120)			Group 2 (*n* = 57)			*p* value
	*n*	%	Mean ± SD	*n*	%	Mean ± SD	
Gender							.020^*∗*^
Male	44	36.7		11	19.3		
Female	76	63.3		46	80.7		
Age (years)			71.41 ± 9.01			73.02 ± 7.79	.249
Weight			66.96 ± 12.46			67.11 ± 9.30	.930
Education level							.436
Preschool	21	17.5		13	22.8		
Elementary/secondary	74	61.7		26	45.6		
High school/vocational	25	20.8		18	31.6		
Surgical site							.904
Left knee	62	51.7		30	52.6		
Right knee	58	48.3		27	47.4		
Smoking history							.035^*∗*^
No	99	82.5		55	96.5		
Yes	11	9.2		1	1.8		
Formerly	10	8.3		1	1.8		
Drinking history							.061
No	102	85.0		54	94.7		
Yes	18	15.0		3	5.3		
Analgesic intake							.965
No	72	60.0		34	59.6		
Yes	48	40.0		23	40.4		
Chronic conditions							.336
No	22	18.3		14	24.6		
Yes	98	81.7		43	75.4		
Surgical experience							.751
No	30	25.0		13	22.8		
Yes	90	75.0		44	77.2		
Anesthetic experience							.938
No	28	23.3		13	22.8		
Yes	92	76.7		44	77.2		
Type of anesthetic							.168
General anesthesia	111	92.5		49	85.9		
Spinal anesthesia	9	7.5		8	14.0		
Average preoperative pain (NRS)			1.89 ± 1.88			1.91 ± 2.17	.964
Highest preoperative pain (NRS)			5.17 ± 2.72			5.16 ± 2.67	.984
Preoperative knee angle			112.37 ± 18.31			112.21 ± 16.45	.956

^*∗*^ *p* < .05.

**Table 2:** Total surgical pain and analgesic belief (*N* = 177).

Variable	Group 1 (*n* = 120)		Group 2 (*n* = 57)		*p* value
	Mean	SD	Mean	SD	
POABS	40.51	7.01	37.88	7.76	.026^*∗*^

Note: POABS = Pain and Opioids Analgesics Beliefs Scale. ^*∗*^*p* < .05.

**Table 3:** GEE analysis of the variation in lowest pain rating in relation to group and time (*N* = 177).

Parameters	Estimated value	Standard error	Wald chi-square	*p* value
Intercept	1.199	.3794	9.979^*∗∗*^	.002
Group				
G_2_ versus G_1_	−.281	.2598	1.189	.275
Time				
T_1_ versus T_0_	3.092	.2492	153.962^*∗∗∗*^	<.001
T_2_ versus T_0_	1.425	.1840	60.003^*∗∗∗*^	<.001
T_3_ versus T_0_	.542	.1810	8.955^*∗∗*^	.003
T_4_ versus T_0_	.258	.1789	2.085	.149
Group × time				
G_2_ T_1_ versus T_0_	−.846	.4100	4.257^*∗*^	.039
versus G_1_ T_1_ versus T_0_				
G_2_ T_2_ versus T_0_	−.021	.3542	.004	.952
versus G_1_ T_2_ versus T_0_				
G_2_ T_3_ versus T_0_	−.243	.3160	.593	.441
versus G_1_ T_3_ versus T_0_				
G_2_ T_4_ versus T_0_	−.065	.3023	.047	.829
versus G_1_ T_4_ versus T_0_				
Gender				
Female versus male	.304	.1370	4.927^*∗*^	.026
Anesthetic method				
General versus spinal	.395	.2239	3.117	.077
POABS	−.011	.0078	1.189	.275

Note: GEE = generalized estimating equations; ^*∗*^*p* < .05;  ^*∗∗*^*p* < .01; ^*∗∗∗*^*p* < .001; G_1_: Group 1; G_2_: Group 2; T_1_: recovery room (day of surgery); T_1_: ward (day of surgery); T_2_: postoperative day 1; T_3_: postoperative day 2; T_4_: postoperative day 3; POABS = Pain and Opioids Analgesics Beliefs Scale.

**Table 4:** Highest pain rating of the two groups during the study (*N* = 177).

Group	Time				
	Day of surgery	Day of surgery	Postoperative	Postoperative	Postoperative
	recovery room	ward	day 1	day 2	day 3
Group 1 (*n* = 120)	3.0	8.1	6.6	5.4	4.5
Group 2 (*n* = 57)	3.1	7.3	6.3	4.8	4.2

**Table 5:** GEE analysis of the highest pain rating variation in relation to group and time (*N* = 177).

Parameters	Estimate value	Standard error	Wald chi-square	*p* value
Intercept	3.266	.6180	27.931^*∗∗*^	<.001
Group				
G_2_ versus G_1_	−.075	.5783	.017	.897
Time				
T_1_ versus T_0_	5.058	.3621	195.092^*∗∗∗*^	<.001
T_2_ versus T_0_	3.558	.3448	106.526^*∗∗∗*^	<.001
T_3_ versus T_0_	2.350	.3337	49.600^*∗∗∗*^	<.001
T_4_ versus T_0_	1.442	.3438	17.589^*∗∗∗*^	<.001
Group × time				
G_2_ T_1_ versus T_0_	−.918	.6386	2.066	.151
versus G_1_ T_1_ versus T_0_				
G_2_ T_2_ versus T_0_	−.400	.6812	.346	.557
versus G_1_ T_2_ versus T_0_				
G_2_ T_3_ versus T_0_	−.648	.6521	.988	.320
versus G_1_ T_3_ versus T_0_				
G_2_ T_4_ versus T_0_	−.319	.6521	.264	.829
versus G_1_ T_4_ versus T_0_				
Gender				
Female versus male	.444	.1978	5.051^*∗*^	.025
Anesthetic method				
General versus spinal	1.124	.3365	11.166^*∗∗∗*^	.001
POABS	−.015	.0122	1.503	.220

Note: GEE = generalized estimating equations; ^*∗*^*p* < .05;  ^*∗∗*^*p* < .01;  ^*∗∗∗*^*p* < .001; G_1_: Group 1; G_2_: Group 2; T_0_: recovery room (day of surgery); T_1_: ward (day of surgery); T_2_: postoperative day 1; T_3_: postoperative day 2; T_4_: postoperative day 3; POABS = Pain and Opioids Analgesics Beliefs Scale.

**Table 6:** Optimal knee flexion angle of the two groups after knee arthroplasty (*N* = 177).

Group	Time		
	Postoperative	Postoperative	Postoperative
	day 1	day 2	day 3
Group 1	75	88	95
Group 2	68	80	90

**Table 7:** GEE analysis of variation in the optimal knee flexion angle in relation to time and group (*N* = 177).

Parameters	Estimated value	Standard error	Wald chi-square	*p* value
Intercept	76.118	18.5774	16.788^*∗∗*^	<.001
Group				
G_2_ versus G_1_	−4.515	3.4833	1.680	.195
Time				
T_1_ versus T_0_	11.600	1.5123	58.832^*∗∗∗*^	<.001
T_2_ versus T_0_	18.307	1.8896	93.862^*∗∗∗*^	<.001
Group × time				
G_2_ T_1_ versus T_0_	−1.696	2.8677	.350	.554
versus G_1_ T_1_ versus T_0_				
G_2_ T_2_ versus T_0_	.492	2.9783	.027	.869
versus G_1_ T_2_ versus T_0_				
Gender				
Female versus male	−4.356	2.4610	3.132	.077
Analgesic intake				
Yes versus no	−1.641	2.3051	.507	.477
Age	.192	.1571	1.502	.220
BMI	−.550	.2948	3.481	.062
Preoperative knee angle	.099	.0659	2.271	.132
Highest painful rating	−1.331	.4637	8.241^*∗∗*^	.004

Note: GEE = generalized estimating equations;  ^*∗∗*^*p* < .01;  ^*∗∗∗*^*p* < .001; G_1_: Group 1; G_2_: Group; T_0_: postoperative day 1; T_1_: postoperative day 2; T_2_: postoperative day 3.

**Table 8:** Anesthetic method of the two groups after knee arthroplasty (*N* = 177).

Variables	Group 1 (*n* = 120)		Group 2 (*n* = 57)		*p *value
	*n*	%	*n*	%	
Anesthetic method					
SA	111	92.50%	49	85.96%	.168
GA	9	7.50%	8	14.04%	

**Table 9:** GEE analysis of the lowest pain rating variation in relation to group and anesthetic method (*N* = 177).

Parameters	Estimate value	Standard error	Wald chi-square	*p* value
Intercept	.895	.124	51.826^*∗∗∗*^	<.001
Time				
T_1_ versus T_0_	2.819	.201	196.65^*∗∗∗*^	<.001
T_2_ versus T_0_	1.418	.158	80.250^*∗∗∗*^	<.001
T_3_ versus T_0_	.463	.149	9.717^*∗∗*^	.002
T_4_ versus T_0_	.237	.145	2.697	.101
Anesthetic method				
GA versus SA	.327	.250	1.712	.191

Note: GEE = generalized estimating equations; ^*∗∗*^*p* < .01; ^*∗∗∗*^*p* < .001; T_0_: recovery room (day of surgery); T_1_: ward (day of surgery); T_2_: postoperative day 1; T_3_: postoperative day 2; T_4_: postoperative day 3.

**Table 10:** GEE analysis of the highest pain rating variation in relation to group and anesthetic method (*N* = 177).

Parameters	Estimate value	Standard error	Wald chi-square	*p* value
Intercept	2.96	.272	118.827^*∗∗∗*^	<.001
Time				
T_1_ versus T_0_	4.763	.300	252.000^*∗∗∗*^	<.001
T_2_ versus T_0_	3.429	.301	129.760^*∗∗∗*^	<.001
T_3_ versus T_0_	2.141	.290	54.420^*∗∗∗*^	<.001
T_4_ versus T_0_	1.339	.286	21.850^*∗∗∗*^	<.001
Anesthetic method				
GA versus SA	1.053	.362	8.459^*∗∗*^	.004

Note: GEE = generalized estimating equations; ^*∗∗*^*p* < .01; ^*∗∗∗*^*p* < .001; T_0_: recovery room (day of surgery); T_1_: ward (day of surgery); T_2_: postoperative day 1; T_3_: postoperative day 2; T_4_: postoperative day 3.

**Table 11:** Optimal knee flexion angle of the two groups after knee arthroplasty by anesthetic method (*N* = 177).

Parameters	Estimate value	Standard error	Wald chi-square	*p* value
Intercept	72.441	1.590	2074.802^*∗∗∗*^	<.001
Time				
T_3_ versus T_2_	12.768	1.266	101.657^*∗∗∗*^	<.001
T_4_ versus T_2_	21.249	1.387	234.793^*∗∗∗*^	<.001
Anesthetic method				
GA versus SA	−2.184	4.552	.230	.631

Note: GEE = generalized estimating equations; ^*∗∗*^*p* < .01; ^*∗∗∗*^*p* < .001; T_2_: postoperative day 1; T_3_: postoperative day 2; T_4_: postoperative day 3.
